# Audiometric and vestibular evaluation in women using the hormonal contraceptive method

**DOI:** 10.1016/S1808-8694(15)30967-8

**Published:** 2015-10-19

**Authors:** Edson Ibrahim Mitre, Alessandra Sousa Figueira, Aparecida Barbosa Rocha, Simone Maria Cordeiro Alves

**Affiliations:** a MD, MS, PhD in Medicine, by the School of Medical Sciences of the Santa Casa – São Paulo, Voluntary Instructor Physician; b Specialist in Audiology by the Center of Specialization in Clinical Speech and Hearing Specialists (CEFAC), Speech and Hearing Specialist - Faculdades Metodistas Integradas Izabela Hendrix; c Specialist in Audiology by the Center of Specialization in Clinical Speech and Hearing Specialists (CEFAC), Speech and Hearing Specialist – Catholic University of Goiás; d MD, Specialist in Mental Health – State University of Montes Claros. Centro de Especialização em Fonoaudiologia Clínica (CEFAC) - Rua Espírito Santo 1892 05018-000 Belo Horizonte MG Tel/fax: (0xx11) 3291-2758

**Keywords:** hearing, contraceptive, hormone, dizziness, tinnitus

## Abstract

**Aim:**

To co-relate the use of hormonal contraceptives with positive Auditory and Vestibular alterations.

**Methods:**

medical history taking, audiometric test and vestibular test was applied to 60 women between the ages of 14 and 35 years old, and 30 of these women are on oral hormonal contraceptive consisting of Estrogen and Progesterone (risk group), for 6 months or more, that had no Auditory or Vestibular complaints prior to the usage of hormones; and 30 women that had never used these hormones (control group), with no Auditory or Vestibular complaints. Medical history was used to select the sample.

**Results:**

Based on otoneurological findings, through quantitative research, we could see the prevailing Irritatative Peripheral Vestibular Syndrome and tinnitus in the risk group, without audiometric alterations.

**Conclusion:**

The use of oral contraceptives can provoke functional alterations in the inner ear, specially tinnitus and Irritative Peripheral Vestibular Syndrome in the risk group; but auditory threshold alterations were not evident.

## INTRODUCTION

Women go through monthly reproductive cycles, which start during puberty and, usually, last during all their reproductive life, stopping at menopause. These cycles prepare the female reproductive tract for pregnancy^1^.

A gonadotrophin release hormone (GnRH), synthesized by the hypothalamus, stimulates the release of two other hormones produced in the pituitary: follicle stimulating hormone (FSH), and the Luteinizing hormone (LH). The former stimulates the development of ovarian follicles and the production of estrogen by the follicular cells, the latter triggers ovulation (acts on the release of the secondary oocyte) stimulating the follicle cells and the corpus luteus to produce progesterone. Estrogen acts specially on the reproductive organs functioning and development regulation; and progesterone acts stimulating endometrial glands to secrete and prepare the endometrium for the blastocyst implantation^1^.

When the oocyte is not fertilized, the corpus luteus starts to degenerate about 10 to 12 days after ovulation; estrogen and progesterone levels drop and the secretory endometrium enters its ischemic phase, and menstruation sets in^1^.

Combined estrogen and progesterone pills (contraceptive pills), taken orally for a period of three weeks have anovulatory action, preventing fertilization from taking place. This occurs through gonadotrophin secretion inhibition by the pituitary acting on the hypothalamus. The progesterone agent present in the pill suppresses LH secretion and the estrogen agent is responsible for the FSH secretion suppression, showing a synergic effect. Besides, the estrogen component enhances the action of the progesterone agents, thus allowing a reduction on the doses of progesterone in the pill composition and, consequently, reducing its adverse effects^2^.

The use of these contraceptive pills by women may cause different adverse reactions, such as: immune, metabolic, nutritional, psychiatric, vascular, ocular, gastrointestinal, hepatobiliary, skin, renal/urinary and auditory alterations; Central Nervous System disorders, and reproductive system disorders. Moreover, the body may trigger vertigo in the pre-menstrual syndrome or during the use of these hormones, due to estrogen and progesterone concentrations^3^, ^4^, ^5^, ^6^, ^7^, ^8^, ^9^, ^10^.

Therefore, when vertigo and/or dizziness/hearing loss appear, it is necessary to investigate prior use of medication; it is necessary to know whether or not the symptoms started with the use of some substance or in changing a medication dosing^11^.

Partial or total loss of auditory or vestibular function, during or after exposure to medications, solvents and other substances is called ototoxicity. Many are the potentially ototoxic substances, including cardiovascular drugs, central nervous system drugs, muscle relaxants, non-hormonal anti-inflammatory, antibiotics, hormones, respiratory tract drugs, antihistamines, contraceptive drugs, cytostatic drugs, anesthetics, appetite moderators, amongst others^12^, ^13^.

Body balance depends on the integrity of the vestibular system, the somatosensorial system and vision. The labyrinth is responsible for the sense of balance and the body's position in space. Dizziness and vertigo set it when something interferes in the normal functioning of these systems.

The biochemical integrity of the inner ear liquids is important for its proper functioning. The hormonal alterations caused by oral hormonal contraceptives may impair the homeostasis of labyrinth fluids, because they have direct influence over the enzymatic processes and on the action of neurtransmitters^14^.

There are some studies that correlate inner ear alterations with the action of hormones, which will be reported below.

The use of oral contraceptives may lower hearing thresholds, without altering stapedial reflex^14^.

There are cases of sudden hearing loss because of the use of estrogen and progesterone, alone or in association. The prolonged use of these hormones may cause sensorineural hearing loss in the high frequencies, besides total or partial vestibular involvement. This is explained when we observe labyrinth irrigation, because the vascular obstruction of the internal auditory artery brings disorders to the territory it irrigates, involving the cochlea, vestibule or both^14^.

Some studies believe that otologic alterations (otosclerosis, hearing loss, progressive hearing loss) in women who use oral hormonal contraceptives may not be related to this therapy alone^15^.

In a study carried out with women who used oral hormonal contraceptive pills, they observed that in relation to hearing capacity and self-perception, this use does not cause significant hearing alterations; however, it favors tinnitus symptoms. Notwithstanding, in another study, women who used oral contraceptives had significant and consistent hearing loss in some frequencies, when compared to normal menstrual cycle women who did not use the pill^16^, ^17^.

This correlation between hearing alterations and the use of oral hormone contraceptives was observed in a case in which a young woman presented with sudden sensorineural hearing loss, preceded by tinnitus, and recovered after treatment. In this case, tinnitus should be considered a warning about the need to interrupt the hormonal therapy^18^.

There are research papers showing that oral contraceptives today have less estrogen and progesterone, being called “low dose pills”. The reduction on the doses of these hormones has significantly reduced sudden hearing loss in those patients^14^.

In order to prove the influence of hormones in the body, normal pregnant women were submitted to vestibular assessment, showing greater sensitivity to mild vestibular stimuli, when compared to the control group (males), thus proving a labyrinth dysfunction during normal pregnancy, specially on the first quarter, probably secondary to the hormonal action^19^, ^20^.

In a study with albino guinea pigs, they studied the effective estrogen action on the hearing, manifested by an increase in brain stem auditory potential thresholds in 47% of the animals, thus confirming the ototoxicity potential of this hormone^21^.

Among causes of vertigo, migraine is predominantly found among teenagers and adults. The use of oral contraceptives, intra-uterine devices or even hysterectomy, should be always considered in cases of recurrent chronic headaches, for there are indications that the use of a contraceptive method, specially hormonal, influence the occurrence of this disease. Among persons with migraine, 81% were female and 77% are between 16 and 50 years^22^.

In this study we shall approach the otoneurological alterations (hearing loss, tinnitus, vestibular symptoms) accruing from the use of hormonal contraceptives by women for a minimum time period of 6 menstrual cycles. Our goal was to correlate the use of oral hormonal contraceptive use with possible hearing and vestibular alterations.

## METHODS

The present study was carried out from the data about patient history, tonal/vocal audiometry and vestibular exam from 30 women who used the oral hormonal contraceptive method and in 30 women who did not use it (control group).

In order to select the sample individuals, some criteria were used: females, aged between 14 and 35 years, minimum of 6 months on oral hormonal contraceptive with estrogen and progesterone, without hearing alterations complaints before starting to use hormonal contraceptive, and without using any other type of medication.

To make up the control group, we selected women aged between 14 and 35 years, who did not use contraceptive hormones nor any other medication, with neither hearing nor vestibular complaints.

Tonal threshold audiometry of all the patients were carried out in a sound proof booth, with the aid of an Amplaid A171 audiometer, calibrated according to the ANSI-69 standard, in 03/19/2003. We assessed the frequencies from 250Hz to 8000Hz (air) and 500Hz to 4000Hz (bone), according to the method used to determine the descending-ascending hearing threshold^2^, ^3^.

The results obtained from the audiometric tests were interpreted as to hearing level, according to the criteria used by Silman & Silverman, and as to the type of hearing loss (mild, moderate, severe, profound)^2^, ^4^.

Vestibular exams were carried out through the Contronic version 5 device. The tests used were: calibration, spontaneous nystagmus (eyes opened and eyes shut), semi-spontaneous nystagmus, positional nystagmus (head torsion to the right, head torsion to the left, pending head), pendular tracking, and post-caloric nystagmus (water at 44º and 30°) (attachment 3).

After data collection, results were statistically treated. We build contingency tables (2 × 2) to describe the hearing and vestibular alterations frequence distribution between the two groups under comparison (risk and control groups). In order to check the significance of this association between the use of oral hormonal contraceptives with hearing and vestibular alterations we used the chi-squared test (c2) at a significance level of a = 0.005. Both the null and alternative hypothesis considered in these tests were, respectively, H0: there is no correlation between the use of oral hormonal contraceptives and hearing and vestibular alterations; and Ha: there is a relation between the use of oral hormonal contraceptive and hearing/vestibular alterations. Since we had a p value below the previously established level of significance a= 0.005), the null hypothesis was rejected (H0), thus showing a significant association between the use of oral hormonal contraceptives and hearing/vestibular alterations.

Ethics: The present study was assessed and approved by the Ethics and Research committee of the Speech Therapy Specialization Clinic (CEFAC) under protocol # 133/04 and it was approved without risk and with the need for a Free and Clarified Informed Consent.

## RESULTS

In the control group, 100% of the sample (30 women) did not complain of hearing loss, tinnitus or dizziness. 70% (21 women) complained of sporadic headaches and 16.7% (5 women), complained of insomnia. In the risk group, 100% of the sample (30 women) did not complain of hearing loss; 33.3% (10 women) complained of tinnitus; 73.3% (22 women) complained of dizziness; 76.7% (23 women) reported sporadic headaches and 23.3% (7 women), insomnia. (Table 1, Table 2, Table 3, Table 4).

**Table 1:** Analysis of tinnitus occurrence in women who used oral hormonal contraception for six months or more (risk group), and in women who had never used it (control group).

		Tinnitus		
Group		Present	Absent	Total
Risk	n	10	20	30
	%	33,3	66,7	100,0
Control	n	0	30	30
	%	0	100,0	100,0
Total	n	10	50	60
	%	16,7	83,3	100,0

x^2^ = 12.00 and p value = 0.001

**Table 2:** Analysis of insomnia occurrence in women who used the oral hormonal contraception method for six months or more (risk group), an in women who had never used it (control group).

		Insomnia		
Group		Present	Absent	Total
Risk	n	7	23	30
	%	23,3	76,7	100,0
Control	n	5	25	30
	%	16,7	83,3	100,0
Total	n	12	48	60
	%	20,0	80,0	100,0

x^2^ = 0.417 and p value = 0.519

**Table 3:** Analysis of the occurrence of sporadic headaches in women who used the oral hormonal contraception method for six months or more (risk group), an in women who had never used it (control group).

		Sporadic headaches		
Group		Present	Absent	Total
Risk	n	23	7	30
	%	76,7	23,3	100,0
Control	n	21	9	30
	%	70,0	30,0	100,0
Total	n	44	16	60
	%	73,3	26,7	100,0

x^2^ = 0.341 and p value = 0.559

**Table 4:** Analysis of dizziness occurrence in women who used the oral hormonal contraception method for six months or more (risk group), an in women who had never used it (control group).

		DIZINESS		
Group		Present	Absent	Total
Risk	n	22	8	30
	%	73,3	26,7	100,0
Control	n	0	30	30
	%	0	100,0	100,0
Total	n	22	38	60
	%	36,7	63,3	100,0

x^2^ = 34.737 and p value = 0.000

In the audiometric test, 100% of the sample (60 women) had audiometric tests within the normal range, compared to 0% of altered audiometric test.

In vestibular testing, the control group showed 23.3% of the sample (7 women) with irritative peripheral vestibular syndrome (IPVS) and 76.7% (23 women) had normal vestibular tests (NVT). In the risk group, 83.3% (25 women) had irritative peripheral vestibular syndrome (IPVS) and 16.7% (5 women) had normal vestibular tests (NVT). (Table 5).

**Table 5:** Vestibular test analysis in women who used the oral hormonal contraception method for six months or more (risk group), an in women who had never used it (control group).

		Vestibular test		
Group		IPVS	NVT	Total
Risk	n	25	5	30
	%	83,3	16,7	100,0
Control	n	7	23	30
	%	23,3	76,7	100,0
Total	n	32	28	60
	%	53,3	46,7	100,0

x^2^ = 21.696 and p value = 0.000

IPVS – irritative peripheral vestibular syndrome; NVT – normal vestibular test.

## DISCUSSION

Based on the results of this research paper, we can see that hearing loss does not seem to be related to the use of oral hormonal contraceptives, since both the risk group and the control group did not show alterations in their audiometric tests, corroborating data from some previous research. Current contraceptive pills are mainly made up of low doses of estrogen and progesterone, thus reducing the occurrence of side effects, specially those related to the auditory function. Notwithstanding, the occurrence of tinnitus in the risk group was significant (c2 = 12.00 and p value = 0.000)^14^, ^15^, ^16^, ^18^.

Results also point that headache and insomnia complaints may not be related to the use of contraceptive medication, because there was no difference between the number of women in the risk group and in the control group who had these symptoms (c2 = 0.341 and p value = 0.559).

Notwithstanding, the headaches reported were identified as sporadic and not as chronic-recurrent (migraine). In some scientific findings, migraines are closely related to the use of hormones^22^.

The use of oral hormonal contraceptive pills may have significantly influenced the complaint of dizziness (c2) = 34.737 and p value = 0.000), since this complaint was not seen in the control group^3^, ^4^, ^5^, ^6^, ^7^, ^8^, ^9^, ^10^, ^11^, ^14^.

The results of the vestibular tests show the relationship between irritative peripheral vestibular syndrome and the use of oral hormonal contraceptives, since 25 in 30 women of the risk group presented IPVS, while only 7 in 30 women of the control group had such alteration (c2 = 21.696 and p value = 0.000). This may be related to the influence of these hormones in the homeostasis of labyrinth fluids^14^.

## CONCLUSION

We then concluded that the use of oral hormonal contraceptives for a period of 6 months or more may cause functional alterations to the inner ear, specially tinnitus and dizziness, the latter is justified by the significant prevalence of irritative peripheral vestibular syndrome in the risk group, but did not show hearing threshold alterations.
